# Tumor immune profiles noninvasively estimated by FDG PET with deep learning correlate with immunotherapy response in lung adenocarcinoma

**DOI:** 10.7150/thno.50283

**Published:** 2020-08-29

**Authors:** Changhee Park, Kwon Joong Na, Hongyoon Choi, Chan-Young Ock, Seunggyun Ha, Miso Kim, Samina Park, Bhumsuk Keam, Tae Min Kim, Jin Chul Paeng, In Kyu Park, Chang Hyun Kang, Dong-Wan Kim, Gi-Jeong Cheon, Keon Wook Kang, Young Tae Kim, Dae Seog Heo

**Affiliations:** 1 Department of Internal Medicine, Seoul National University Hospital, Seoul, Republic of Korea.; 2 Department of Thoracic and Cardiovascular Surgery, Seoul National University Hospital, Seoul, Republic of Korea.; 3 Department of Nuclear Medicine, Seoul National University Hospital, Seoul, Republic of Korea.; 4 Division of Nuclear Medicine, Department of Radiology, Seoul St. Mary's Hospital, College of Medicine, The Catholic University of Korea, Seoul, Republic of Korea.; 5 Cancer Research Institute, Seoul National University, Seoul, Republic of Korea.

**Keywords:** Immunotherapy, tumor microenvironment, fluorodeoxyglucose positron emission tomography, deep learning, gene expression profile

## Abstract

**Rationale:** The clinical application of biomarkers reflecting tumor immune microenvironment is hurdled by the invasiveness of obtaining tissues despite its importance in immunotherapy. We developed a deep learning-based biomarker which noninvasively estimates a tumor immune profile with fluorodeoxyglucose positron emission tomography (FDG-PET) in lung adenocarcinoma (LUAD).

**Methods:** A deep learning model to predict cytolytic activity score (CytAct) using semi-automatically segmented tumors on FDG-PET trained by a publicly available dataset paired with tissue RNA sequencing (n = 93). This model was validated in two independent cohorts of LUAD: SNUH (n = 43) and The Cancer Genome Atlas (TCGA) cohort (n = 16). The model was applied to the immune checkpoint blockade (ICB) cohort, which consists of patients with metastatic LUAD who underwent ICB treatment (n = 29).

**Results:** The predicted CytAct showed a positive correlation with CytAct of RNA sequencing in validation cohorts (Spearman rho = 0.32, *p =* 0.04 in SNUH cohort; spearman rho = 0.47, *p =* 0.07 in TCGA cohort). In ICB cohort, the higher predicted CytAct of individual lesion was associated with more decrement in tumor size after ICB treatment (Spearman rho = -0.54, *p <* 0.001). Higher minimum predicted CytAct in each patient associated with significantly prolonged progression free survival and overall survival (Hazard ratio 0.25, *p =* 0.001 and 0.18, *p =* 0.004, respectively). In patients with multiple lesions, ICB responders had significantly lower variance of predicted CytActs (*p =* 0.005).

**Conclusion:** The deep learning model that predicts CytAct using FDG-PET of LUAD was validated in independent cohorts. Our approach may be used to noninvasively assess an immune profile and predict outcomes of LUAD patients treated with ICB.

## Introduction

Lung adenocarcinoma (LUAD) is one of the most benefitted cancers in the era of cancer immunotherapy according to several landmark clinical trials supporting the use of immune checkpoint blockades (ICB), anti-programmed cell death 1 (PD-1) antibody and anti-programmed cell death ligand 1 (PD-L1) antibody 1-3. Many of these clinical trials used PD-L1 immunohistochemistry (IHC) expression scores as a biomarker to predict responders to ICB. Although the higher PD-L1 IHC expression level is associated with better response rates, the level alone is unsatisfactory for clinicians to refer to as a sole biomarker 4. In this regard, other potential biomarkers, including tumor mutation burden and tumor-infiltrating lymphocytes, have been introduced, and integration of these mechanism-based biomarkers has been suggested to accurately predict the response to ICB 5,6.

However, tissue-based biomarkers have limitations to unleash the complexity of inter-tumoral heterogeneity, since they mostly rely on a single tumor sample from approachable lesion in practice. Furthermore, immune-escape mechanism would evolve dynamically during anti-cancer treatment; thus, it is difficult to identify the current status of immune profiles from an archival sample 7,8. Also, each of metastatic lesions would confer unique immunogenic profiles, derived from various factors including clonal heterogeneity and tumor microenvironment, and result in different genomic/transcriptomic signature, even in a single patient 9-12. This spatial heterogeneity of the tumor microenvironment (TME) causes atypical responses to ICB, such as simultaneous development of a new lesion despite regression of tumor burden 13. These atypical and variable responses are unpredictable before the treatment using such tissue-based biomarkers of a small subset of the tumor.

Therefore, to facilitate the understanding in the dynamics of all metastatic tumors in real-world clinical practice, noninvasive methods to assess the immune landscape of tumors are necessary. This may be achieved through the application of radiomics incorporating medical images and deep learning technologies to mine novel data that are associated with clinical events 14. Fluorodeoxyglucose positron emission tomography (FDG-PET) is widely used to identify primary and all metastatic tumors of the whole body at once. As FDG-PET reflects the metabolic features of the tumor, which are highly associated with the tumor immune microenvironment 15-18, the pattern and heterogeneity of FDG uptake in the tumor could be related to immune profiles of the TME.

In this study, we developed and validated a deep learning-based biomarker to predict an immune profile of the TME using FDG-PET and RNA sequencing (RNA-seq) data of LUAD. We used cytolytic activity score (CytAct) to represent the immune profile of TME, as CytAct is associated with cytotoxic CD8+ T cell activity against tumor and is simple to calculate 19. We also tried to demonstrate if our deep learning model can be applied to predict not only response to ICB of individual lesions, but also overall response and survival of corresponding patients.

## Methods

### Training cohort and validation cohort

To develop a deep learning model for estimating an immune profile in the TME of LUAD, we used a publicly available dataset, The Cancer Imaging Archive (TCIA) dataset 20, defined in this study as a Stanford cohort 21. The FDG PET imaging data was downloaded from TCIA 20, and RNA-seq data was downloaded from Gene Expression Omnibus (GEO) (http://www.ncbi.nlm.nih.gov/geo/) under accession number GSE103584 21.

To validate this model, two cohorts were used; one from our center (SNUH cohort) 22, and the other from The Cancer Genome Atlas (TCGA cohort). Patients with both FDG-PET and RNA-seq data were only included in each cohort. The presurgical imaging data of SNUH cohort were retrospectively collected and the RNA expression dataset of the SNUH cohort was available at GEO under accession number GSE40419 22. The imaging data and RNA expression data of TCGA LUAD were downloaded from TCIA 20 and cbioportal 23, respectively.

**Figure 1:**
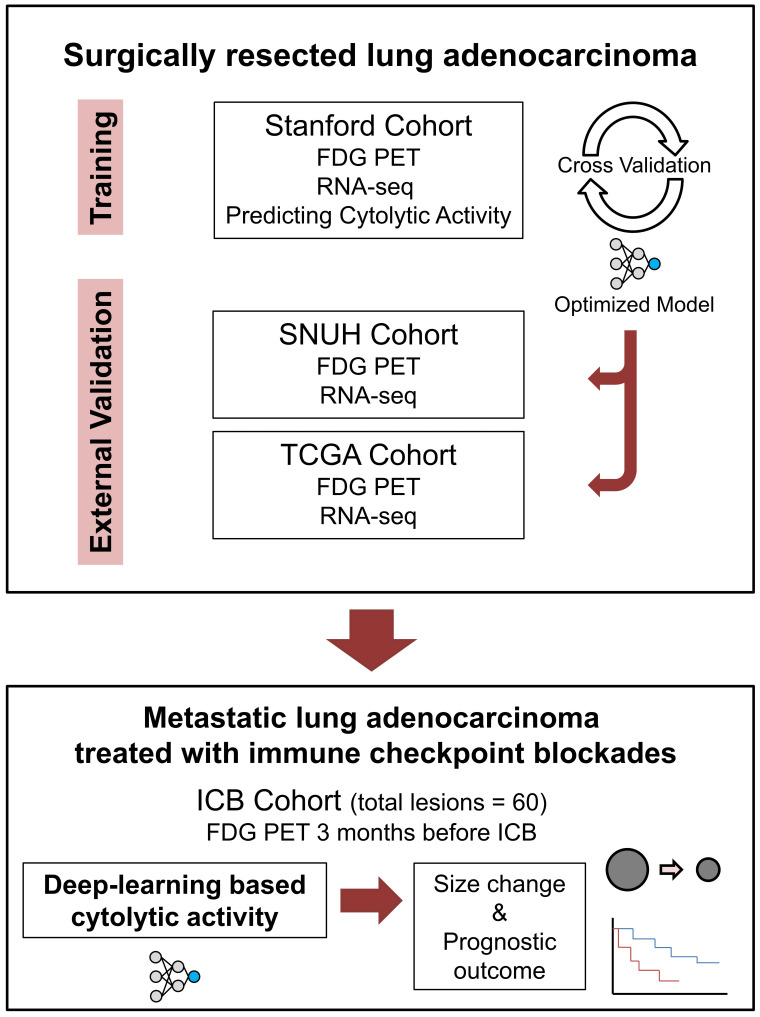
** Schematic flow charts of overall study design.** Deep learning model was trained using Stanford cohort (n = 93) with RNA-seq and FDG-PET images to predict CytAct. After cross validation and optimization, the model was then applied to two independent external validation cohorts; SNUH cohort (n = 43), and TCGA cohort (n = 16). To further demonstrate whether the CytAct predicted by our model correlates with response to ICB and survival outcome, the model was applied to ICB cohort (n = 29), which consists of metastatic lung adenocarcinoma treated with ICBs. Abbreviations: FDG PET, fluorodeoxyglucose positron emission tomography; ICB, immune checkpoint blockade.

The ICB cohort consisted of the metastatic LUAD patients who received ICB monotherapy outside the clinical trial setting and underwent FDG PET evaluation within 3 months before the start of ICB. Patients with no measurable lesion by Response evaluation criteria in solid tumors (RECIST) or no available response evaluation were excluded 13. Demographics and treatment history of ICB cohort patients were retrospectively reviewed. Lesions were measured according to RECIST 1.1, and response evaluation was done according to iRECIST criteria 13,24. All lesions were reviewed by 2 medical oncologists (C.Y.O. and C.P.) and 1 nuclear medicine physician (H.C.) and underwent thorough discussion on the consensus of measures. Flow chart for overall study design is summarized in **Figure 1.**

### Choosing target gene expression signature

CytAct is defined by the mean value of the expression of granzyme A (*GZMA*) and perforin 1 (*PRF1*) normalized by z-score, which is easy to calculate with RNA-seq data 19. Although CytAct is known to represent cytotoxic CD8+ T cell activity against tumor 19, we first evaluated whether CytAct would represent the immune profile of TME in the training cohort by analyses on correlations of CytAct with interferon-γ related profile (IFNG score) and Immune score by xCell tool 25,26. IFNG score was calculated by the mean value of the expression of 6 genes (*IDO1*, *CXCL9*, *CXCL10*, *IFNG*, *HLA-DRA*, and *STAT1*) 25. Immune score was calculated by using the xCell tool (http://xcell.ucsf.edu/) 26. The RNA-seq data of the training cohort was log 2 normalized before these analyses.

### Deep learning model for predicting immune profiles

**Figure 2:**
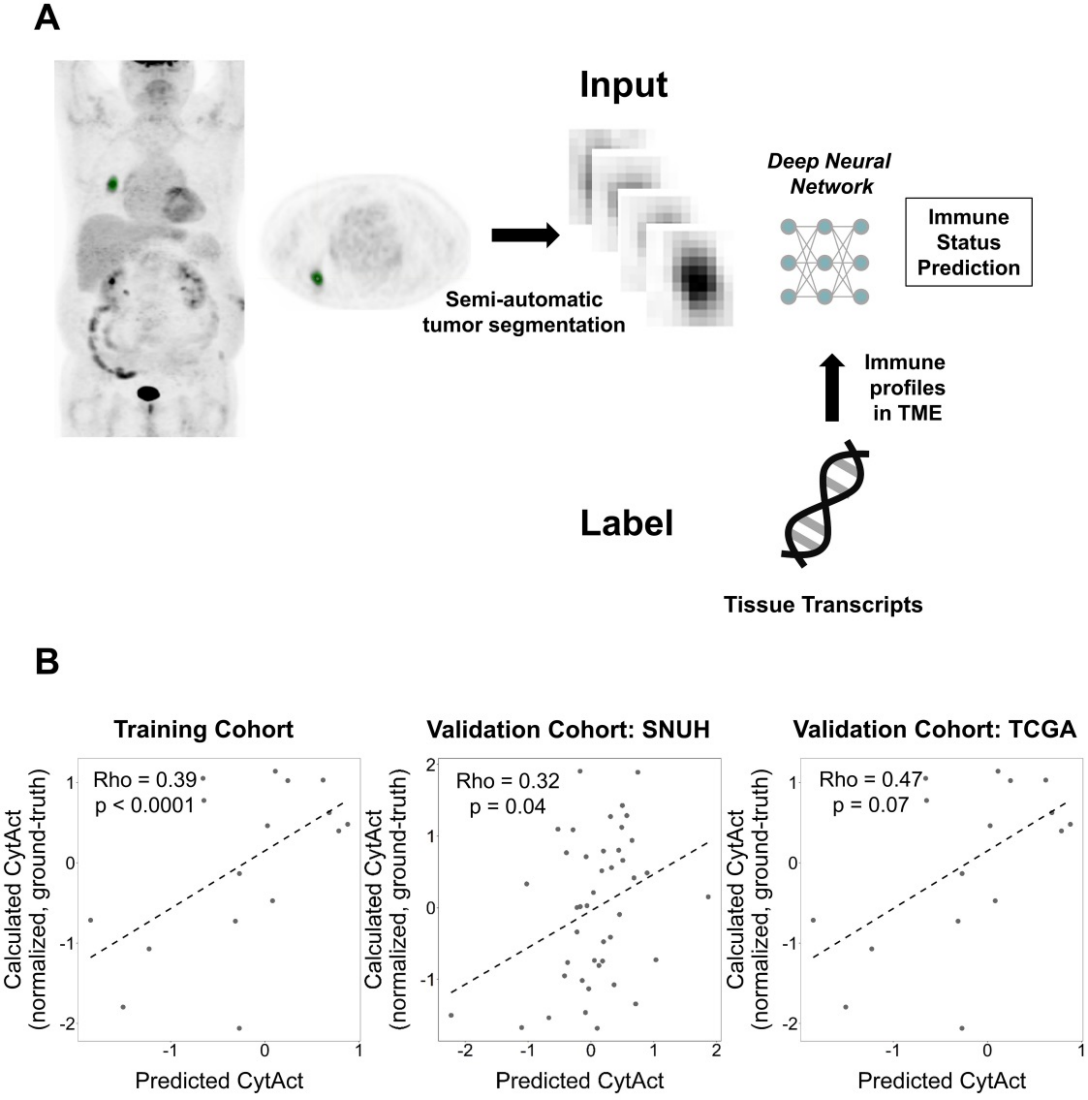
** Generation of the deep learning algorithm.** (**A**) An overview of the process of generating a deep learning algorithm to predict immune profiles of a tumor. The FDG-avid primary lung cancer lesion was identified and semi-automatically segmented. This segmented tumor was used as an input of a deep neural network. The target outputs, immune profiles, were cytolytic activity score (CytAct) and immune cell enrichment scores as representative immune profiles of the tumor microenvironment (TME) calculated from RNA-seq data. (**B**) Plots showing a Spearman correlation of predicted CytAct and calculated CytAct to demonstrate the performance of our deep learning algorithm prediction. In each plot, the X-axis represents predicted CytAct, and the Y-axis represents normalized calculated CytAct based on RNA-seq. From left to right, each plot was drawn for the training cohort (Spearman rho value 0.39 and *p <* 0.0001), our independent cohort with both presurgical imaging and RNA-seq data (Spearman rho value 0.32 and *p =* 0.04), and the TCGA cohort (Spearman rho value 0.47 and *p =* 0.07), respectively. The dashed line in each plot is a regression line. Abbreviations: TME, tumor microenvironment.

The overall approach for the development of the deep learning model is summarized in **Figure 2A.** A primary LUAD mass was visually identified and semi-automatically segmented using an adaptive threshold-based method 27. This tumor segmentation process was performed on LIFEx software (ver 4.0.0, www.lifexsoft.org) 28. More specifically, the threshold of tumor was defined by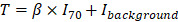, where *I_70_* is the mean uptake value of voxels with an uptake greater than 70% of the maximum uptake and *I_background_* is the mean uptake value of background voxels. We set the parameter β = 0.3 29. A 3D cube-shaped volume that included a segmented tumor lesion was used as an input for the deep learning model. The architecture of the model was based on the 3-dimensional convolutional neural network (**Figure S1**) 30. To overcome the limited number of training data (n = 93), image augmentation using random rotations was performed. More specifically, image augmentation was aimed to develop a robust deep learning model against the rotation of tumors which can be affected by position and location of tumors. Thus, we generated random numbers between 0 and 90 degrees and rotated the segmented tumor for three-axes. For the training process, fifteen volumes of randomly rotated tumors were generated for each iteration of the training. Such type of image augmentation is commonly used in the training of deep learning for natural images as well as medical images 31,32. The target output was cytolytic activity score (CytAct), defined by the expression of granzyme A (*GZMA*) and perforin 1 (*PRF1*) normalized by z-score 19. 10-fold cross-validation was applied for the training cohort and the two external validation cohorts were not used until the model was optimized by the training/internal validation sets of the cross-validation. More detailed methods for FDG-PET image acquisition, tumor segmentation and deep learning model generation are available in **Supplementary Methods.**

### Validation of the deep learning model

FDG PET volumes of the segmented tumor were acquired from two independent cohorts with the same method applied for the training set and used as inputs of the model to predict CytAct. These predicted CytAct was compared with CytAct calculated by the RNA-seq. For immune cell enrichment analysis, the xCell tool (http://xcell.ucsf.edu/) 26 was employed, and the RNA-seq dataset of each cohort was used as input. Hierarchical clustering with distance calculation by Pearson correlation coefficient was used to cluster results of immune cell enrichment analysis.

### Deep learning-based CytAct estimation in the ICB cohort

The deep learning model was applied to the ICB cohort, patients with metastatic LUAD who underwent ICB treatment. The whole-body FDG PET image before the ICB treatment was used as an input. We selected target lesions on the baseline scan by the RECIST 1.1 criteria 13. The minimum size of the lesion chosen was 1.5 cm in the short axis for the lymph node and 1 cm in the long axis for all other lesions. Responders were defined as patients who experienced partial response (PR) and nonresponders were defined as patients who did not.

### Statistical analysis

To evaluate the correlation of two continuous variables, Spearman correlation was used. To compare differences of continuous variables between groups, two-sided Wilcoxon rank-sum test was used. Univariate and multivariate logistic regression analysis were used to determine whether predicted CytAct and PD-L1 IHC levels were significantly associated with size changes of the lesions. To evaluate the performance of predicted CytAct in discriminating responders and nonresponders, univariate logistic regression was performed and area under curve (AUC) from receiver operating characteristic curve was calculated. For survival analysis, the Kaplan-Meier method and log-rank test was used. A p-value less than 0.05 was considered as statistically significant. All statistical analysis was performed with R 3.5.0 (https://www.r-project.org/).

### Ethics

All data of the SNUH and ICB cohort were collected and analyzed after approval of the institutional review board (No. 1810-149-983) and in accordance with the declaration of Helsinki.

## Results

### Correlation of CytAct with other immune expression profiles in the training cohort

The CytAct estimated by RNA-seq in the training cohort significantly correlated with both IFNG score and Immune score (Spearman rho = 0.50, *p <* 0.001 and Spearman rho = 0.66, *p <* 0.001, respectively, **Figure S2**). These findings indicate that CytAct may represent the tumor immune profiles in the training cohort.

### Prediction of immune profiles of LUAD by deep learning application to PET images

A total of 93 patients with LUAD had both RNA-seq data and FDG PET in Stanford cohort. The validation cohorts included 43 patients in SNUH cohort and 16 patients in TCGA cohort. The deep learning based predicted CytAct was positively correlated with the CytAct estimated by RNA-seq in the training cohort and this correlation was validated in the two validation cohorts (**Figure 2B**). Notably, the predicted CytAct of the training cohort was a pooled result of internal validation sets using cross-validation. In case of conventional features represented by FDG PET, standardized uptake value (SUV) showed significant but modest correlation with RNA-seq based CytAct or predicted CytAct in the training cohort but not in the validation cohorts, and metabolic tumor volume (MTV) did not correlate with either CytAct or predicted CytAct at all (**Figure S3**). In the meanwhile, the predicted and RNA-seq based CytAct positively correlated with the enrichment of effector immune cells, including CD8+ T-cells in the TME (**Figure S4**). These findings supported that the deep learning model captures patterns related to immune profiles apart from conventional FDG PET features.

### ICB response prediction using deep learning based CytAct in metastatic LUAD

**Figure 3:**
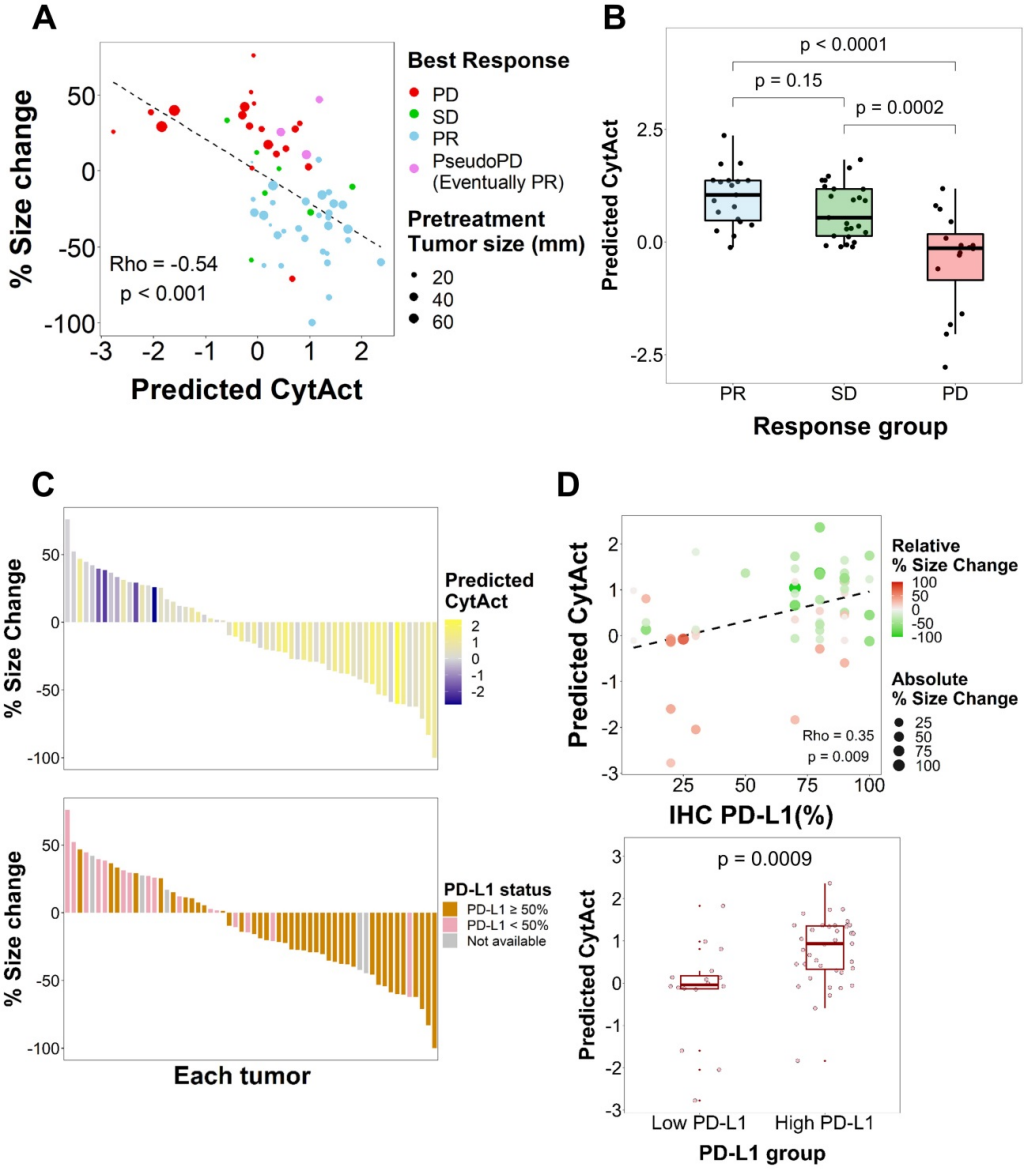
** Predicted CytAct estimated by each tumor lesion on baseline FDG PET before ICB.** (**A**) The relationship between predicted CytAct and the percentage (%) size change of each lesion was analyzed. Each dot represents a lesion. The color of dots was determined by the best response of each patient on the ICB treatment. The size of dots represents pretreatment tumor size. Predicted CytAct and percentage size change showed a Spearman rho correlation of -0.54 (*p <* 0.001). The regression line is shown with a dashed line. (**B**) A boxplot shows the comparison of the predicted CytAct of each response group. The boxplots depict median, upper quartile and lower quartile values with horizontal segments and 1.5× interquartile range with a vertical segment. The response group was determined by the best response of each patient. Predicted CytAct values were significantly different between the PD group and other groups by two-sided Wilcoxon rank-sum test (PD vs SD, *p =* 0.0002; PD vs PR, *p <* 0.0001; PR vs SD, *p =* 0.15). (**C**) A waterfall plot was arranged by size changes in individual lesions. Note that each bar on the same X-axis on upper and lower plots represents the same lesion. The bars in the upper plot were colored according to predicted CytAct, while those in the lower plot were colored according to PD-L1 IHC status obtained by patientwise evaluation. Note that the lesions evaluated for PD-L1 IHC status were different from the lesions of predicted CytAct, as most PD-L1 IHC data were obtained from archival tissue samples or a representative tumor among multiple lesions. (**D**) A relationship between predicted CytAct and PD-L1 IHC percentage was presented (Spearman rho 0.35 and *p =* 0.009). In the upper plot, each dot represents a lesion. The size and color of a dot represents absolute and relative percentage tumor size change, respectively. The dashed line represents a regression line. The lower plot is a boxplot according to PD-L1 IHC status, defined as 'high PD-L1' if PD-L1 IHC percentage is 50% or more and low if otherwise. The boxplot depicts median, upper quartile and lower quartile values with horizontal segments and 1.5x interquartile range with a vertical segment. The high PD-L1 group had a significantly higher predicted CytAct by two-sided Wilcoxon rank-sum test (*p =* 0.0009). Abbreviations: IHC, immunohistochemistry; PD, progressive disease; PD-L1, programmed cell death ligand 1; PR, partial response; SD, stable disease.

**Figure 4:**
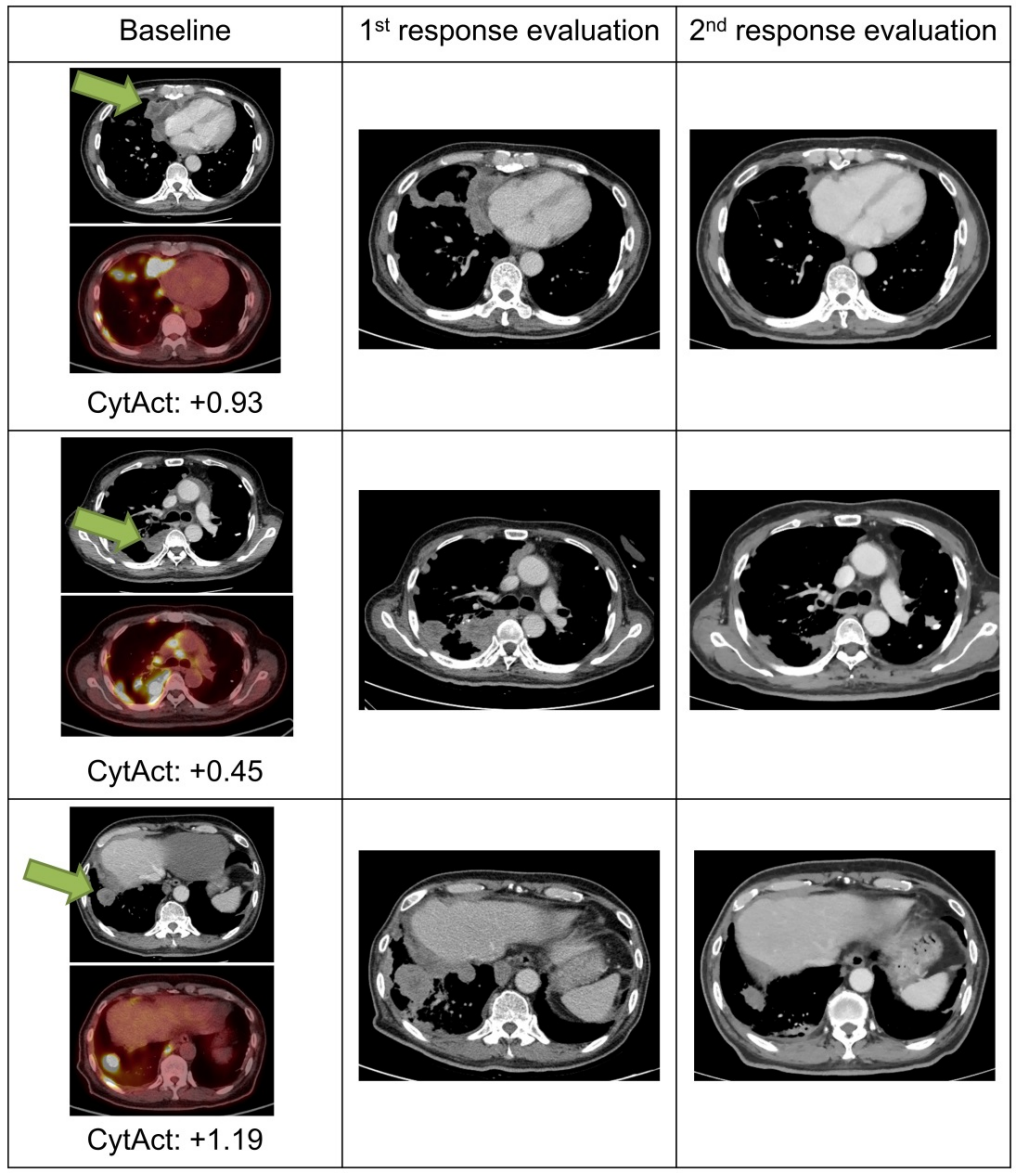
** Images and predicted CytAct of a pseudo-progression case.** A 69-year-old female patient diagnosed with LUAD underwent 2 cycles of pemetrexed and cisplatin, after which the cancer progressed as shown in the images of the baseline column. The patient then received pembrolizumab, and 1^st^ response evaluation was done after 1 month. Although the 1^st^ evaluation CT scan showed progression, the patient continued pembrolizumab because the general condition of the patient was clinically improving. After 2 more months, a 2^nd^ response evaluation CT scan was done and showed regression of tumor masses. The target lesion is marked with green arrows in the figure. Predicted CytAct values as shown below the PET images were higher than 0.107, the value that discriminates responders from nonresponders described later in the manuscript.

As both multiple metastatic and primary tumor lesions can be noninvasively assessed by whole-body FDG PET images, the model was applied to each tumor lesion of patients in ICB cohort to predict the CytAct. A total of 29 patients with 60 tumor lesions were analyzed by the deep learning-based model. Demographics of the patients available in **Table 1** and characteristics of the lesions available in **Table 2.** In these lesions, median predicted CytAct was 0.48, ranging from -2.78 to 2.36. The size change of tumor lesions after the ICB treatment showed significant negative correlation with the predicted CytAct (**Figure 3A**). Notably, a patient who showed pseudo-progression and eventually experienced PR had lesions with the predicted CytAct higher than the median value (**Figure 3A, Figure 4**). Tumor lesions of the patients with progressive disease (PD) showed significantly lower predicted CytAct than those of patients with PR (**Figure 3B**). A waterfall plot showed higher predicted CytAct was associated with decreased size of each tumor lesion (**Figure 3C**).

To evaluate whether the predicted CytAct provides additional predictive value to PD-L1 expression, another waterfall plot was drawn with PD-L1 IHC expression percentage (**Figure 3C**). It also showed higher PD-L1 IHC expression was associated with decreased lesion size. Of note, PD-L1 IHC expression was evaluated by one representative lesion or archived primary tumor sample that was different from metastatic tumor lesions. The predicted CytAct showed a weak positive correlation with PD-L1 expression level (**Figure 3D**). Multivariate logistic regression showed that the predicted CytAct was significantly associated with the size change of each lesion independent from PD-L1 IHC expression level (*p =* 0.001 for the predicted CytAct and *p =* 0.051 for PD-L1).

As aforementioned that PD-L1 IHC was obtained from a tissue that was different from lesions evaluated for the predicted CytAct, we next sought to evaluate the predicted CytAct with available PD-L1 expression level for exactly corresponding lesions. A total of 9 lesions from 9 patients were available for the analysis on the exactly corresponding lesions. Despite the small number of samples, it showed a tendency that size change seemed to be associated with the predicted CytAct more than PD-L1 expression level. There was only weak correlation between predicted CytAct and PD-L1 expression level (**Figure S5**).

### Deep learning-based CytAct as a biomarker for predicting outcome of patients treated with ICB

**Figure 5:**
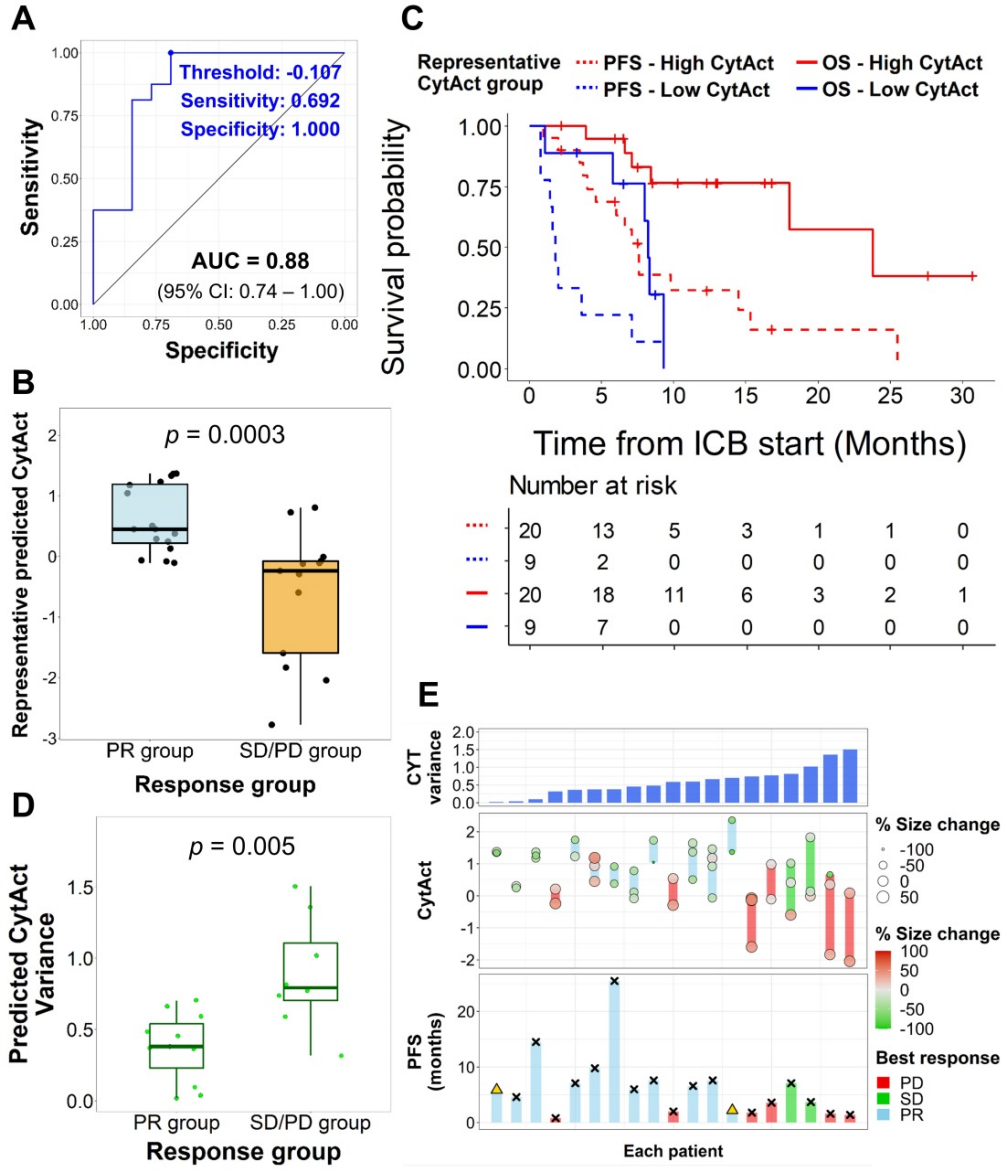
** Association of predicted CytAct with clinical outcomes.** (**A**) A receiver operating characteristics curve shows the performance of minimum predicted CytAct of a patient in predicting whether a patient showed partial response (PR) to immunotherapy (AUC = 0.88, 95% CI: 0.74 -1.00). The best value discriminating the PR group is shown by a blue dot (Representative CytAct -0.107, sensitivity and specificity at which 0.692 and 1.000, respectively). (**B**) A boxplot shows a comparison of minimum predicted CytAct (representative predicted CytAct) according to the response group by two-sided Wilcoxon rank-sum test (*p =* 0.0003). The boxplots depict median, upper quartile and lower quartile values with horizontal segments and 1.5x interquartile range with a vertical segment. (**C**) Kaplan-Meier survival curves represent progression-free survival (PFS, dashed lines) and overall survival (OS, solid lines) of immunotherapy according to the representative predicted CytAct group. The high and low representative predicted CytAct was determined according to the threshold value depicted in Figure 5A. High representative predicted CytAct was significantly associated with prolonged PFS (HR: 0.25 and 95% CI: 0.10-0.62) and OS (HR: 0.18 and 95% CI: 0.05-0.67). Censored data are marked with cross segments and numbers at risk are demonstrated on the table at the bottom. (**D**) A boxplot shows differences between responders (PR group) and nonresponders (SD/PD group) in terms of variance of predicted CytAct. The nonresponder group had significantly higher variance of predicted CytAct by two-sided Wilcoxon rank-sum test (*p =* 0.005), implicating that higher heterogeneity in the predicted CytAct was associated with nonresponse. The boxplots depict median, upper quartile and lower quartile values with horizontal segments and 1.5x interquartile range with a vertical segment. (**E**) Three plots share the same X-axis with each corresponding to an individual patient. The patients were arranged by CytAct variance in increasing order as shown in the upper bar plot. The middle plot consists of all predicted CytAct values, size changes of each lesion and best response harbored by each patient. The lower plot shows PFS of each patient to immunotherapy. Bars marked with 'x' depict the progression, and a triangle depicts censored data. Abbreviations: AUC, area under curve; CI, confidence interval; CYT, cytolytic activity score; HR, hazard ratio; PFS, progression free survival; OS, overall survival; PD, progressive disease; PR, partial response; SD, stable disease.

Although the predicted CytAct of each tumor lesion showed association with the response to ICB, patient-wise response evaluation is needed to determine whether a patient would benefit from treatment with ICB. Therefore, we sought to evaluate whether the predicted CytAct also correlates with progression-free survival (PFS) and overall survival (OS). Since 19 patients had multiple predicted CytAct from multiple lesions, we first tried to define a representative predicted CytAct for each patient. We hypothesized that one among the minimum, mean or maximum value of predicted CytAct from multiple lesions of a patient would be suitable for a representative predicted CytAct. When we compared how well those 3 variables discriminate responders from nonresponders, the minimum predicted CytAct performed significantly (*p =* 0.021 by univariate logistic regression analysis) and the best (AUC of minimum predicted CytAct 0.88, 95% CI: 0.74-1.0 vs. AUC of mean and maximum predicted CytAct 0.84, 95% CI: 0.70-0.98 and 0.74, 95% CI: 0.54-0.93, respectively, **Figure 5A**, **Figure S6**). Therefore, we used the minimum predicted CytAct as a representative CytAct of each patient.

The representative CytAct was significantly higher in patients who experienced PR (*p =* 0.0003, **Figure 5B**). We divided the patients into 2 groups, high CytAct group and low CytAct group, by the representative CytAct cutoff of -0.107, the optimal point according to the receiver operating characteristic curve in **Figure 5A**. The high CytAct group had significantly longer PFS and OS (*p =* 0.0012, HR: 0.25, 95% CI: 0.10-0.62 and *p =* 0.0042, HR: 0.18, 95% CI: 0.05-0.67, respectively, **Figure 5C**). The AUC of predicted CytAct in discriminating responders and nonresponders in subsets of patients who received nivolumab or pembrolizumab were 0.81 and 1.0, respectively. For the patient who received atezolizumab, the representative CytAct was -0.121 and the best response was SD.

We additionally analyzed whether the intratumoral heterogeneity of the CytAct of each patient was associated with the outcome. More specifically, we sought to demonstrate whether the predicted CytAct can depict the immune heterogeneity in 19 patients with multiple lesions. The degree of heterogeneity in each patient was calculated by a variance of the predicted CytAct. Notably, nonresponders had significantly higher heterogeneity in predicted CytAct compared to responders (*p =* 0.005, **Figure 5D**), and univariate logistic regression analysis showed a higher degree of heterogeneity was significantly associated with nonresponse to ICB (*p =* 0.04). The overall predicted CytAct distribution, response and PFS of the 19 patients are demonstrated in **Figure 5E**. When the 19 patients were divided into high and low heterogeneity groups by the median value, the high heterogeneity group had a tendency of shorter PFS and OS (*p =* 0.075, HR: 2.67, 95% CI: 0.89-7.97; and, *p =* 0.06, HR: 3.66, 95% CI: 0.87-15.38, respectively, **Figure S7**).

## Discussion

We developed a deep learning model to predict CytAct using a noninvasive whole-body image, FDG PET. The predicted CytAct was validated by showing a positive correlation with RNA-seq data in the independent validation cohorts. When we applied our model to patients who underwent ICB, the predicted CytAct showed association with the size change in each lesion including pseudo-progression. Although the correlations and associations were weak, these findings implicate that by utilizing larger number of cases with PET and RNA sequencing, the approach to predict CytAct by PET may be a potentially feasible method for noninvasive estimation of the immune microenvironment of individual tumor lesions.

Interestingly, we found that the predicted CytAct had consistencies with observations in clinical assessments. The finding that the minimum predicted CytAct best represented the response and survival of a patient may be ascribed to criteria used for the response and survival measure, in this case, iRECIST 13. The minimum predicted CytAct as a representative biomarker for each patient implicates that the least immunogenic lesion may be responsible for prognosis and ultimately need to be targeted for survival prolongation, consistent with the concept that the clone that survives the selective pressure from anti-tumor effects eventually becomes the main clone and progresses 33. Additionally, higher heterogeneity in predicted CytAct among multiple lesions was associated with poor outcomes in our study, which may be interpreted as the higher heterogeneity of immune profiles implicates the higher chance of a resistant clone, which will soon affect patient outcomes.

Recently, many multi-omics studies have enlightened the complex relationship between the tumor and immune microenvironment. Despite the comprehensive understandings of the tumor immune microenvironment, bringing these results to clinical practice is hurdled by requirements for large resources and limited available biopsy samples. In particular, it is controversy whether a single biopsy truly represents all the characteristics of the tumor as genomic, transcriptional and immunologic heterogeneity in metastatic lesions may result in ICB response heterogeneity 12,34. Previously reported deep learning model predicting tumor-infiltrating lymphocytes by evaluation of the TME in histology also have a limitation of the requirement of tumor biopsy 35. Therefore, PET-based ICB response prediction has strengths in being noninvasively applicable and reproducible in the clinic and reflecting immunogenic features of all metastatic lesions, allowing us to evaluate multiple tumor lesions in the current state of a patient and in the serial follow-up. In this regard, the noninvasive assessment may potentially impact the current clinical practice of ICB treatment by supporting conventional biomarkers based on the tissue biopsy.

Our model can be applied to FDG PET data of various institutions. Technically, the inputs of our model are voxels of FDG PET images rather than manually extracted features. Although a few papers have tried to develop an algorithm to predict immune profiles of the tumor using texture features 36,37, which are secondary quantitative values estimated by the relationship between voxels, these features have intrinsic limitations in repeatability and standardization 38,39. Our model is more reliable and reproducible, as it used a semi-automatic tumor segmentation followed by normalization with uptake values and matching voxel sizes for the input of the model. These processes can minimize possible variations between centers, machines, and image reconstruction methods. For the convenience of readers, we provided a demo application on a website (https://fdgpetdlimmune.appspot.com) to use our model predicting CytAct from FDG PET data.

Our deep learning model estimated the immune profiles by identifying patterns of tumor metabolism, although it is hardly demonstrable of how the model predicts CytAct from FDG PET. This issue regarding interpretability is a limitation of recently developed artificial intelligence models applied to medical imaging 14,40. A few studies that attempted to predict responders to ICB by machine learning algorithms trained by comparing responding and nonresponding lesions 36,37 also had such an issue. Nonetheless, growing evidence of the association between metabolic profiles and immune profiles of the TME 15 support our model that FDG uptake patterns could be varied by the profiles of the TME. In addition, metabolic interactions that occur in tumors and the microenvironment are becoming recognized as important in the anti-tumor immune reaction 16. A previous study on FDG uptake patterns in primary and secondary lymphoid organs to stratify patients who receive immunotherapy also implicate potential role of FDG-PET in recognizing immune reaction in a patient 41. Thus, we could assume that neural networks look at these complex metabolic profiles and interactions to define CytAct as a whole, though we should solve the issue of interpretability in the future.

There are limitations in our study. First, this study is a retrospective study with a small number of samples and involves CytAct which is not validated as a biomarker for ICB response. Due to the small number of samples, the correlations of predicted CytAct with RNA-seq in the training and validation cohorts were weak, and power to discriminate responders and nonresponders was not sufficient. For example, there were lesions with score around 0 and broad range of size changes from high increase to high decrease (**Figure 3A**). Therefore, the accuracy of our model needs to be improved with larger prospective cohorts with both FDG PET and RNA-seq data to be applied real world clinical practice. Second, although we found that the predicted CytAct performed better than PD-L1 IHC status, more comparison by evaluating corresponding lesions is required since only 9 matched lesions were available in our study. In addition, all patients in the ICB cohort had positive PD-L1 IHC, which is due to a national insurance policy that only allows ICB for PD-L1 positive LUAD. Therefore, it would be valuable to further validate our model in PD-L1 negative tumors. Similarly, whether our model to train an algorithm predicting CytAct and immunotherapy response would work in cancer types other than LUAD is another potential research question to be addressed. In addition, the performance of our model based on each of different ICBs further need to be explored for clinical applicability. Nonetheless, our findings still demonstrated a possible method to bridge basic research and clinical practice and a potential biomarker that can be obtained by widely used FDG PET without any additional harm to patients.

## Conclusion

In conclusion, we developed a potentially feasible predictive imaging biomarker using a deep learning model to predict CytAct and immunotherapy response. Our approach may be used to noninvasively assess an immune profile and predict outcomes of LUAD patients treated with ICB. Further studies to improve the performance of our model with larger prospective cohorts and to comprehensively evaluate predicted CytAct with other immune profiles such as PD-L1 IHC and CD8 T cell infiltrate are warranted.

## Supplementary Material

Supplementary figures and tables.

## Figures and Tables

**Table 1:** Demographics of patients included in the study who received ICB

	Number (%)
Age	Median 64 (Range 38-92)
**Sex**	
Male	24 (82.8)
Female	5 (17.2)
**PD-L1 status**	
Positive, ≥ 50%	17 (58.6)
Positive, 1~50%	9 (31.0)
Negative	0 (0)
NA	3 (10.3)
**Received ICB**	
Pembrolizumab	10 (34.5)
Nivolumab	18 (62.1)
Atezolizumab	1 (3.4)
**ICB administered line of treatment**	
1^st^	5 (17.2)
2^nd^	15 (51.7)
3^rd^ or more	9 (31.0)
**Molecular study**	
*EGFR* exon 19 deletion	1 (3.4)
*EGFR* exon 20 insertion	1 (3.4)
*ALK* translocation	3 (10.3)
Others^a^	3 (10.3)
None	21 (72.4)
**Best response**	
PR	16 (55.2)
SD	3 (10.3)
PD	10 (34.5)

a: Other genomic alterations include *KRAS* G13D, *RET* translocation, *MET* exon 14 skipping mutation. Abbreviations: ICB, immune checkpoint blockade; NA, not available; PD, progressive disease; PD-L1, programmed cell death ligand 1; PR, partial response; SD, stable disease.

**Table 2:** Characteristics of 60 tumor lesions in ICB cohort

	Number (%)
**Type of mass**	
Primary	13 (21.7)
Metastatic	47 (78.3)
**Site of mass**	
Lung	20 (33.3)
Lymph node	17 (28.3)
Adrenal gland	7 (11.7)
Bone (Osteolytic lesion with soft tissue component)	6 (10.0)
Mediastinum	4 (6.7)
Pleural nodule	3 (5.0)
Soft tissue	3 (5.0)

## Abbreviations

AUC: area under curve
CI: confidence interval
CytAct: cytolytic activity score
FDG-PET: fluorodeoxyglucose positron emission tomography
GEO: gene expression omnibus
HR: hazard ratio
ICB: immune checkpoint blockade
IHC: immunohistochemistry
LUAD: lung adenocarcinoma
MTV: metabolic tumor volume
OS: overall survival
PD: progressive disease
PD-1: programmed cell death 1
PD-L1: programmed cell death ligand 1
PFS: progression-free survival
PR: partial response
RECIST: Response evaluation criteria in solid tumors
RNA-seq: RNA sequencing
SD: stable disease
SNUH: Seoul National University Hospital
SUV: standardized uptake value
TCGA: The Cancer Genome Atlas
TCIA: The Cancer Imaging Archive
TME: tumor microenvironment
