# Supplementary material for: Tumor immune profiles noninvasively estimated by FDG PET with deep learning correlate with immunotherapy response in lung adenocarcinoma
Source: Theranostics. 2020 Aug 29;10(23):10838–48. doi: 10.7150/thno.50283 (PMC7482798; doi:10.7150/thno.50283)
Supplement: Supplementary file 1 — Supplementary figures and tables. [file thnov10p10838s1.pdf]

SUPPLEMENTARY METHODS

FDG PET imaging

FDG PET imaging was acquired according to the clinical routine protocol of our center. A dose of 5·18 MBq/kg of FDG was intravenously injected after patients fasted for > 6 hours before the PET imaging. The emission scan was acquired 60 minutes after the FDG injection on dedicated PET/CT scanners (Biograph mCT40 and mCT64, Siemens Healthcare, Germany). After the emission scan, a CT scan was consecutively obtained for attenuation correction. PET images were reconstructed using ordered-subset expectation maximization with an iteration number of 2 and 21 subsets. The reconstructed image matrix size was 256 × 256. FDG PET data of the Stanford and TCGA datasets were acquired according to the standard protocols of each center.

Tumor segmentation

A spherical volume-of-interest was drawn to include target tumor lesion for each patient. As a semiautomatic tumor segmentation, an adaptive threshold of SUV was used to delineate the margin [1]. This tumor segmentation process was performed on LIFEx software (ver 4.0.0, www.lifexsoft.org) [2]. Then, we set a 3-dimensional bounding box to include the segmented tumor. To consider the pattern of the tumor margin, two voxels of 6-directions (two directions for x, y, and z dimensions each) were additionally padded to the bounding box. This 3-dimensional cube volume was used as an input for the model. As the matrix size of FDG PET is different according to the reconstruction algorithm, the segmented cube-shaped volume was resliced to have the same voxel size, 4·0 mm^3.

Training of the deep learning model

The segmented tumor was an input for the deep learning model. The cube-shaped volume that included the segmented tumor and padding described above was scaled by using a simple equation with standardized uptake value (SUV): $C_{voxel,i} = (SUV_{voxel,i} - 0.5)/20$. Because the size of input images was variable, a convolutional neural network was designed to produce outputs regardless of the matrix size of the input. More specifically, two convolutional layers with 16 and 32 convolutional kernels were followed by a global max pooling layer [3]. The activation function of two convolutional layers was tanh (**Figure S1**). The global max pooling layer produced 32-dimensional feature vectors regardless of the input size. In addition, 128-dimensional feature vectors were produced by a fully connected layer. The target output was cytolytic activity score (CytAct) [4], defined by the expression of granzyme A (*GZMA*) and perforin 1 (*PRF1*) normalized by z-score. We used a 10-fold cross-validation using pairs of the segmented tumor and RNA-seq of Stanford cohort (n = 93) to optimize the training of the deep learning model by determining training parameters and model architectures. Furthermore, to overcome the limited number of data, image data of the training set were augmented by randomly rotating segmented tumors with 3 axes for each iteration of the training except the internal validation set selected by the cross-validation. The segmented tumor images of the two external validation sets (SNUH and TCGA cohorts) were not used until the model was optimized by the training/internal validation sets. The loss function of the neural network was defined by mean-absolute-error between predicted immune profiles, i.e. the CytAct and those estimated by RNA-seq. To train the model, an ADAM optimizer was used. The number of epochs for iterative training was set as 50.

References

1. Nestle U, Kremp S, Schaefer-Schuler A, et al. Comparison of different methods for delineation of 18F-FDG PET-positive tissue for target volume definition in radiotherapy of patients with non-Small cell lung cancer. J Nucl Med. 2005; 46: 1342–8.

2. Nioche C, Orlhac F, Boughdad S, et al. Lifex: A freeware for radiomic feature calculation in multimodality imaging to accelerate advances in the characterization of tumor heterogeneity. Cancer Res. 2018; 78: 4786–9.

3. Zhou B, Khosla A, Lapedriza A, Oliva A, Torralba A. Learning Deep Features for Discriminative Localization. In: Proceedings of the IEEE conference on computer vision and pattern recognition. 2016.

4. Rooney MS, Shukla SA, Wu CJ, Getz G, Hacohen N. Molecular and genetic properties of tumors associated with local immune cytolytic activity. Cell. 2015; 160: 48–61.

SUPPLEMENTARY FIGURES

Figure S1.

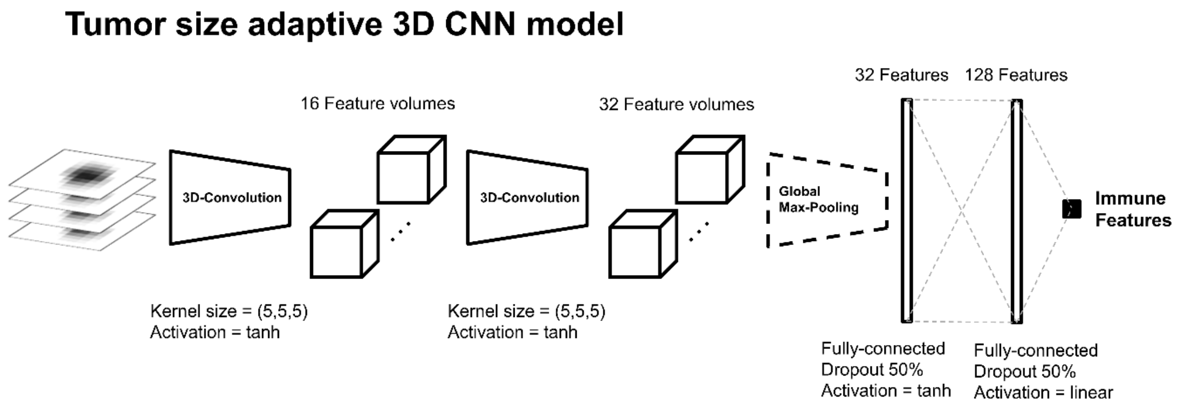

Figure S1. Tumor size adaptive 3D CNN model

Figure depicting the 3D CNN model for the deep learning algorithm used in this study. Two 3-dimensional convolutional layers were applied to the input volume, which included the segmented tumor lesion. After the two convolutional layers, a global max-pooling layer was applied to extract feature vectors. A fully connected layer was followed, and then the hidden features were connected to the output of CytAct estimated by RNA-sequencing data.

Figure S2.

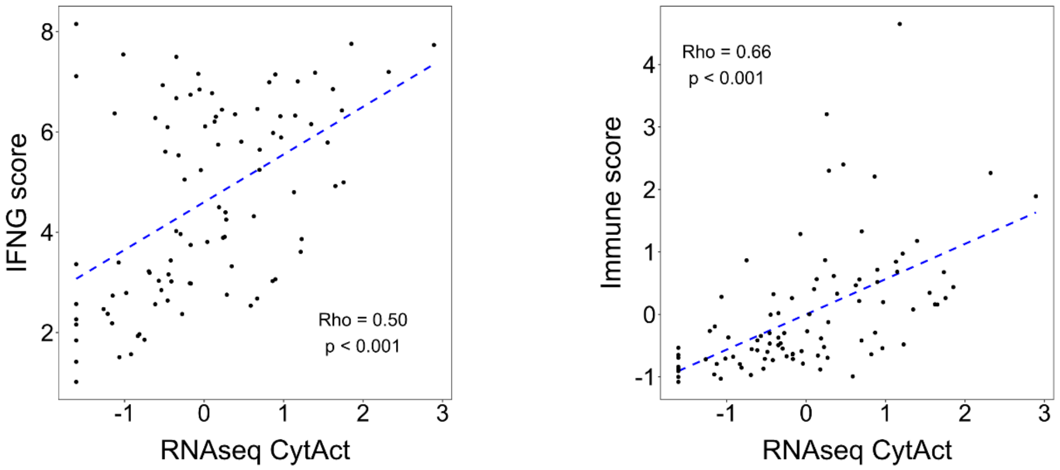

Figure S2. Correlation of RNA-seq based CytAct with IFNG score and Immune score in the training cohort

Scatterplots depict correlation of RNA-seq based CytAct with IFNG score (left) and Immune score (right). Each dot represents each sample in the training cohort. The blue dashed line represents regression line in each plot. RNA-seq based CytAct significantly correlated with both IFNG score (spearman rho = 0.50, $p < 0.001$) and Immune score (spearman rho = 0.66, $p < 0.001$), implicating that CytAct may represent tumor immune profiles in the training cohort.

Figure S3.

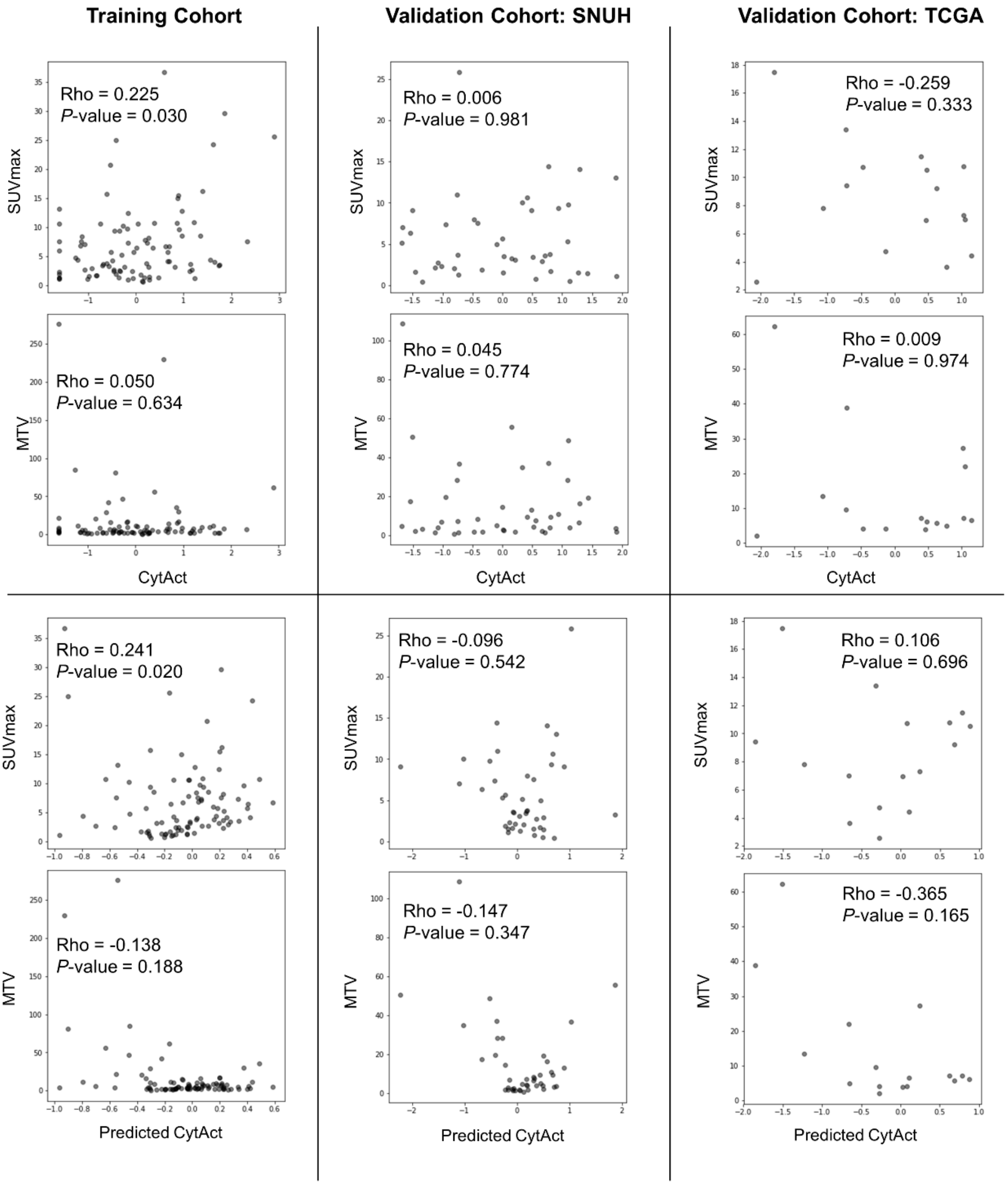

Figure S3. Relationships of CytAct with conventional PET parameters

The upper 6 plots show relationships between calculated CytAct from RNA sequencing data and either maximum standardized uptake value (SUV) or metabolic tumor volume (MTV) in each cohort. The lower 6 plots show relationships between predicted CytAct values and either

maximum SUV or MTV in each cohort.

Figure S4.

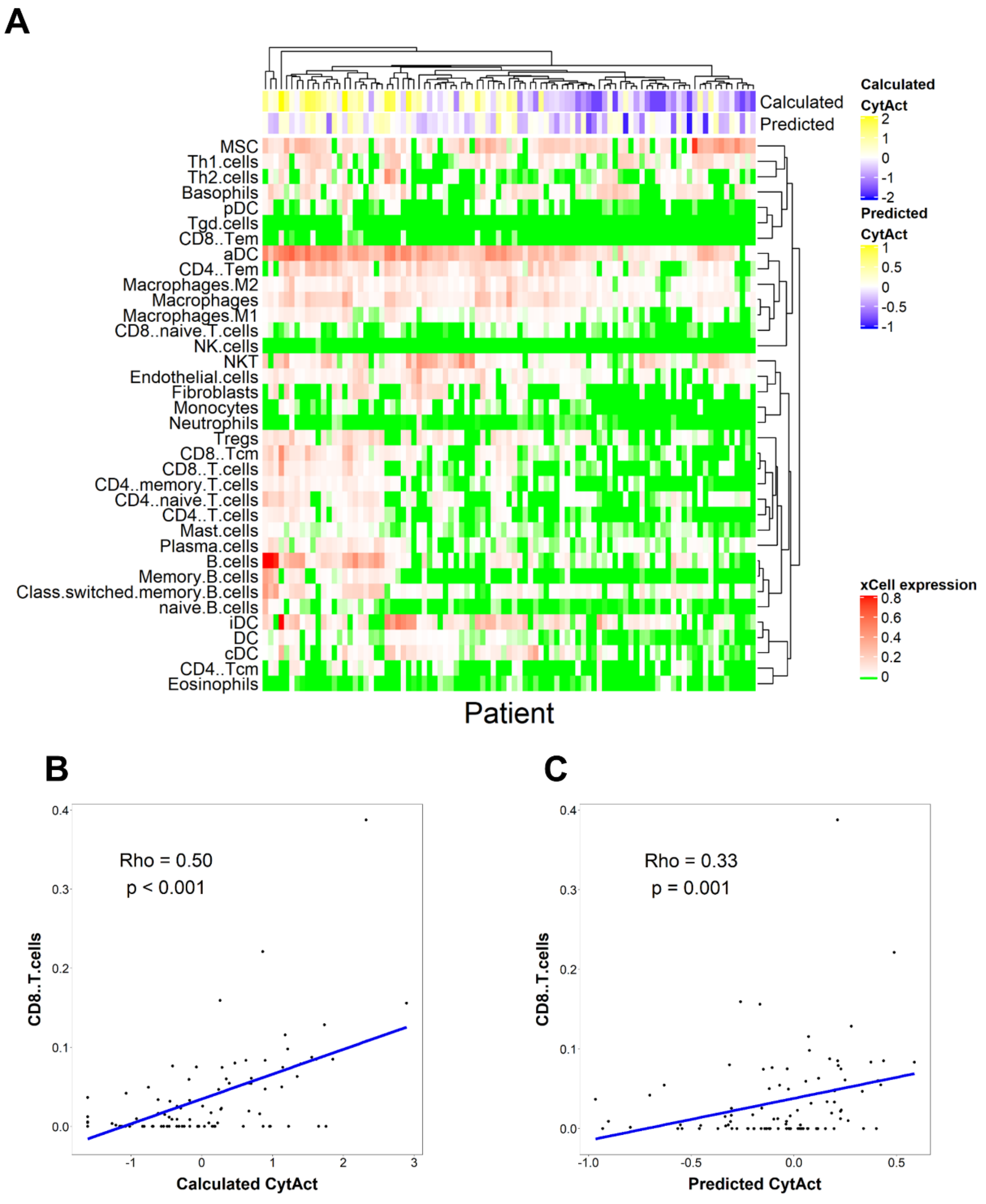

Figure S4. Predicted CytAct in correlation with immune cell profiles of the tumor microenvironment

(A) Heatmap showing immune cell enrichment analysis. The immune cell enrichment analysis results are expressed as xCell expression. Each column represents each patient and

each row represents the immune cell type as annotated. On the top of the heatmap, calculated CytAct by RNA-sequencing data (Calculated CytAct) and deep learning-predicted CytAct (Predicted CytAct) are shown. (B, C) Correlation of Calculated CytAct (Predicted CytAct) values and CD8+ T cell enrichment values (Spearman rho 0.50, $p < 0.001$ for calculated CytAct and spearman rho 0.33, $p = 0.001$ for predicted CytAct).

Figure S5.

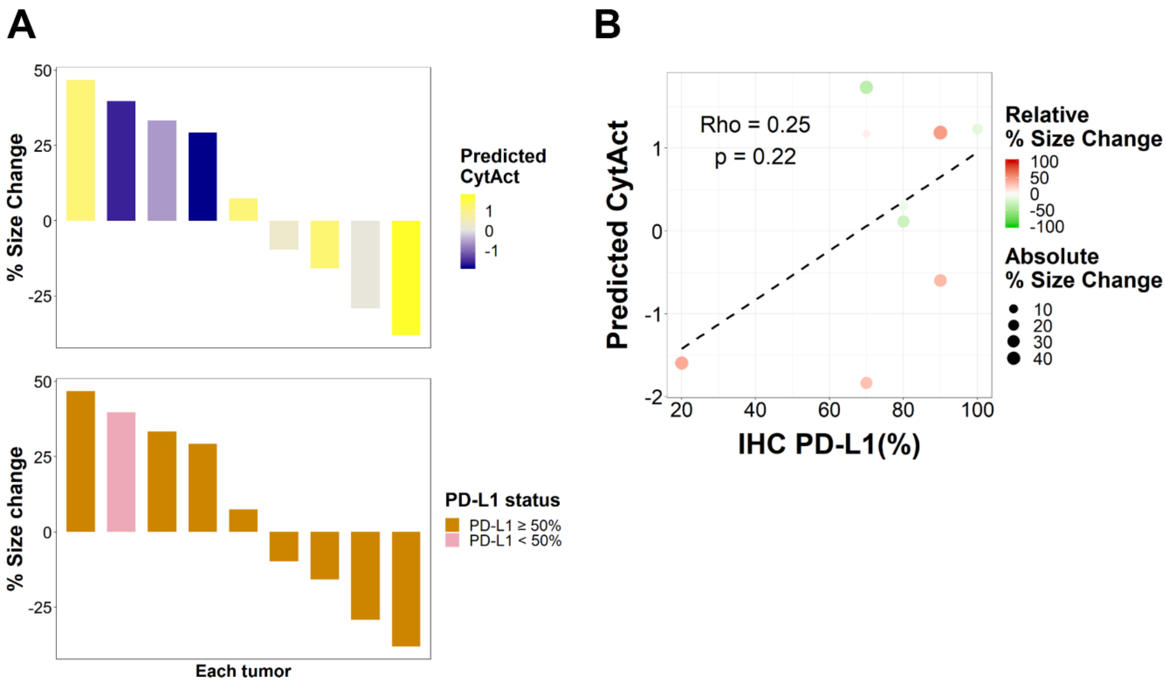

C

Patient index	Best Response	Predicted CytAct	Size change	PD-L1 IHC percentage (%)
Pt1	PR	1.732305	-38.0508	70
Pt2	PR	0.114989	-29.1975	80
Pt3	PD	-1.8343	29.2561	70
Pt4	PR	1.185487	46.77955	90
Pt5	PD	-1.59576	39.66852	20
Pt6	PR	0.301685	-9.72198	80
Pt7	SD	-0.59624	33.33333	90
Pt8	SD	1.231139	-15.7974	100
Pt9	PR	1.172799	7.427165	70

Figure S5. The relationship between predicted CytAct and PD-L1 IHC percentage in matched lesions

(A) Waterfall plot arranged by size changes in each lesion. Each bar on the same X-axis on upper and lower plots represents the same lesion. The bars in the upper plot were colored

according to predicted CytAct, yellow being higher and blue being lower, while those in the lower plot were colored according to PD-L1 IHC status of the original patient. (B) The dot plot shows correlation of PD-L1 IHC percentage and predicted CytAct (Spearman rho 0.25 and $p = 0.22$). Each dot represents a lesion. The size and color of a dot represents absolute and relative percentage size change, respectively. The dashed line shows the regression line. (C) A table showing value of predicted CytAct, PD-L1 IHC percentage, size change and response.

Figure S6.

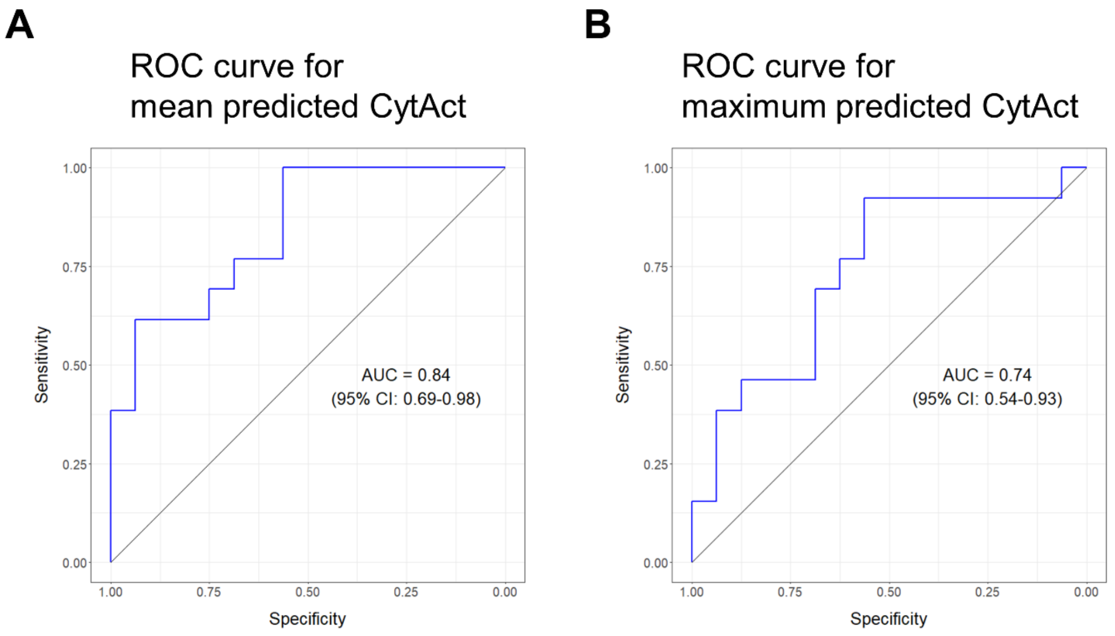

Figure S6. Performance of other representative CytAct values in predicting response to immunotherapy.

(A, B) ROC curve showing performance of mean and maximum predicted CytAct of a patient in determining whether a patient showed PR to immunotherapy (AUC = 0.84, 95% CI 0.69 – 0.98 for mean predicted CytAct and AUC = 0.74, 95% CI 0.54 – 0.93 for maximum predicted CytAct). AUC, area under curve; CI, confidence interval

Figure S7.

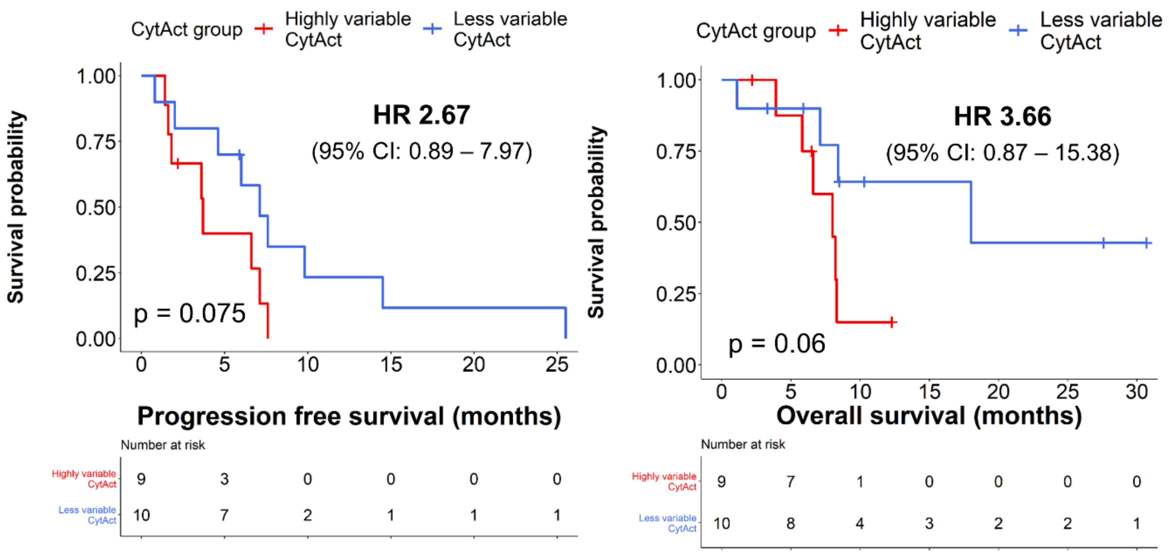

Figure S7. Kaplan-Meier survival curves showing PFS and OS of immunotherapy according to the predicted CytAct heterogeneity group.

The patients were divided into high and low variable groups by median value of predicted CytAct variances. Highly variable predicted CytAct tended to associate with shorter PFS (HR 2·67 and 95% CI 0·89 – 7·97) and OS (HR 3·66 and 95% CI: 0·87 – 15·38). Censored data are marked with cross segments and numbers at risk are demonstrated on each table.
